# Escherichia coli Necrotizing Fasciitis After Abdominoplasty Combined With Liposuction: A Report of a Case and Review of the Literature

**DOI:** 10.7759/cureus.86261

**Published:** 2025-06-18

**Authors:** Charbel B Aoun, Nancy Zeaiter, Elie Moukawam, Majd Hassan, Walid Hreibeh

**Affiliations:** 1 Plastic and Maxillofacial Surgery, Al-Zahraa Hospital University Medical Center, Beirut, LBN; 2 Plastic and Reconstructive Surgery, Lebanese University Faculty of Medicine, Beirut, LBN; 3 Plastic and Reconstructive Surgery, Lebanese Hospital Geitaoui, University Medical Center, Achrafieh, LBN; 4 Plastic and Reconstructive Surgery, Lebanese University, Beirut, LBN; 5 General Medicine, Lebanese University Faculty of Medicine, Beirut, LBN

**Keywords:** abdominoplasty, case report, escherichia coli, infective complications, liposuction, necrotizing fasciitis

## Abstract

Abdominoplasty and liposuction rank among the most common aesthetic plastic surgeries and are considered safe procedures, although intraoperative and postoperative complications can occur. Hematoma, seroma, infections, deep venous thrombosis, and death are some of the encountered complications. Necrotizing fasciitis is a rare, severe, and deadly soft-tissue infection that can develop in healthy patients who undergo common aesthetic operations such as abdominoplasty or liposuction. Early detection and treatment of these complications are crucial. This report documents a rare case of monomicrobial *Escherichia coli* necrotizing fasciitis after abdominoplasty combined with liposuction. Factors associated with a lower risk of complications include being a healthy non-smoker of normal weight, aged under 55 years, having a shorter interval between bariatric and cosmetic surgery, ensuring proper hand hygiene, and exercising extreme caution during abdominal flap undermining. Exceptionally rare cases of *E. coli* infections following abdominoplasty or liposuction were described in the literature. Several hypotheses can explain the *E. coli* infection that can complicate these surgeries, such as poor hand hygiene in some patients, contamination during perioperative care, delayed pre-processing cleaning of surgical instruments and the adhesive property of the instruments, post-sterilization contamination, and the translocation hypothesis. This report underscores the importance of early recognition and prompt management of necrotizing fasciitis, even in low-risk patients undergoing common aesthetic procedures.

## Introduction

While abdominoplasty ranks among the most common aesthetic plastic surgeries, boasts high patient satisfaction, and is considered a safe procedure, its intraoperative and postoperative complications can pose significant challenges for surgeons [1].

Necrotizing fasciitis (NF) is a soft-tissue infection that can develop in healthy patients who undergo common aesthetic operations, such as abdominoplasty or liposuction, which can appear as an ecchymosis visible after surgery, and which can progress rapidly to septic shock and death if left untreated [2]. It was first described by Hippocrates in the fifth century [3]. It is usually related to bacterial invasion of the fascia, quickly spreading to muscles, subcutaneous fat, and overlying skin areas [4]. It affects nearly 0.4 in every 100,000 people per year in the United States [5] and 0.13% of patients who undergo liposuction [6]. Overall infections (including NF) tend to occur in 27.2% of all cases of abdominal operations, including abdominoplasty alone, and abdominoplasty combined with liposuction or body contouring. Combined procedures, in particular abdominoplasty with liposuction, significantly increase the risk of complications such as infections (3.8%), as compared to abdominoplasty alone (3.1%) [7]. Multiple procedures realized simultaneously, obesity, tobacco use, and poor hand hygiene represent important risk factors for developing postoperative abdominal infection [7-9]. The most common isolated germs in infections encountered after abdominoplasty are mainly the inhabitants of the normal skin bacterial flora, regrouping *Staphylococcus aureus*, *Staphylococcus epidermidis*, and *Streptococcus pyogenes* [8].

We hereby report a case of a 33-year-old female who had an abdominoplasty combined with liposuction, after which she developed an NF, caused by a quite uncommon pathogen (*Escherichia coli*), requiring an inpatient aggressive treatment with multiple surgical debridement and intravenous antibiotics.

This case report was prepared in accordance with the CARE (Case Report) guidelines to ensure standardized and complete reporting. Written informed consent was obtained from the patient for publication of this case report and any accompanying images.

## Case presentation

A 33-year-old female patient, previously healthy, light smoker, with a history of two cesarean sections and sleeve gastrectomy (five years before the presentation), with an estimated body mass index (BMI) of 28 kg/m², was admitted electively to Al-Zahraa Hospital University Medical Center, Lebanon for abdominoplasty and liposuction of flanks and the abdomen (total of 1.5 liters). Thirty minutes before the incision, she was given 2 g intravenous (IV) cefazolin. Her initial postoperative course was smooth and uncomplicated, and she was discharged home on amoxicillin/clavulanic acid 1 g twice a day for one week.

**Figure 1:**
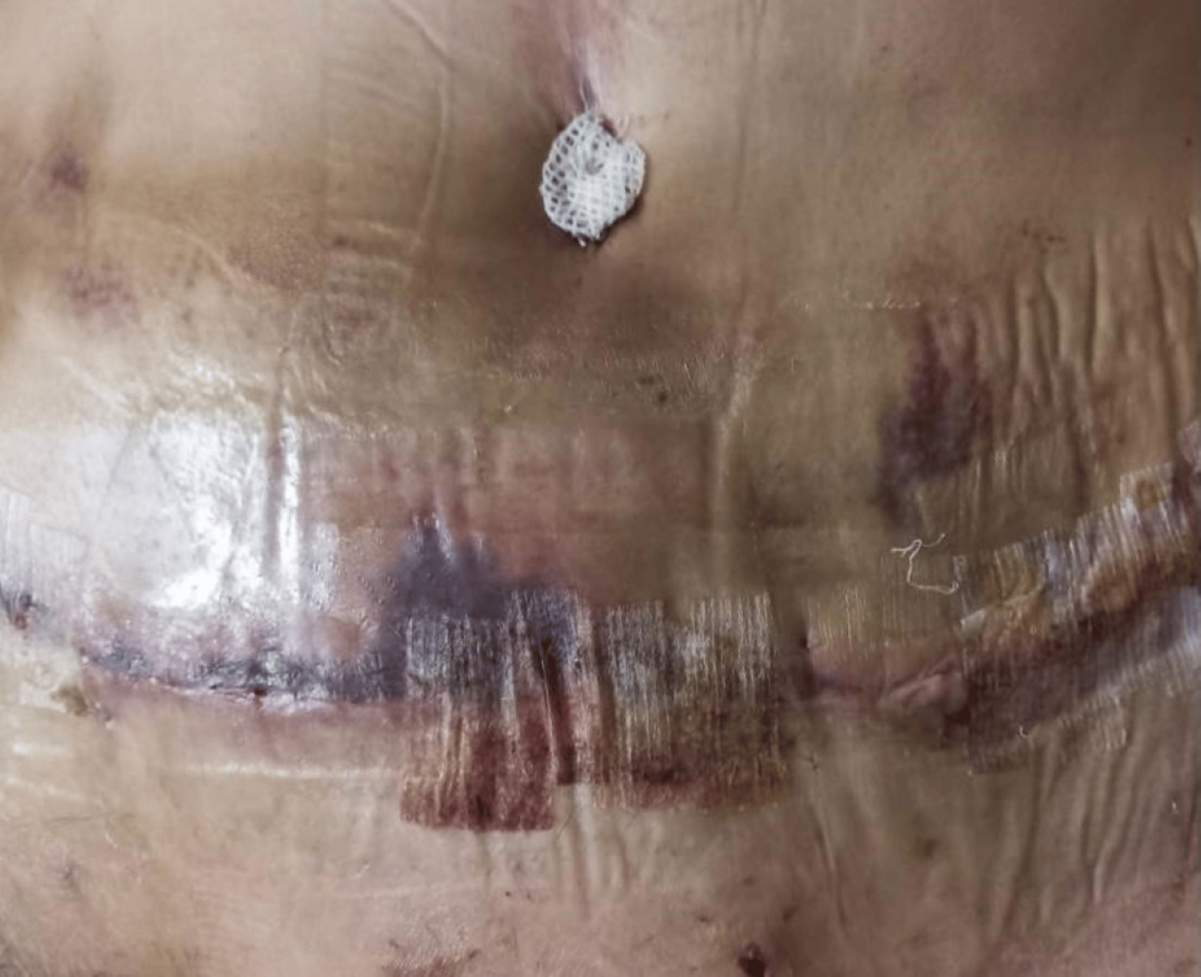
Inspection of the abdomen immediately at admission, showing ecchymosis and signs of ischemia/necrosis.

On postoperative day nine at night, the patient called us complaining of fever and chills. She denied any abdominal pain or oozing from the surgical site. She tested negative for coronavirus disease 2019 (COVID-19) by polymerase chain reaction (PCR), so we asked her to present to the hospital to investigate a possible surgical infection. The physical exam showed the following: febrile patient, ecchymosis and erythema below the umbilicus, and a soft, non-tender abdomen (Figure 1).

**Figure 2:**
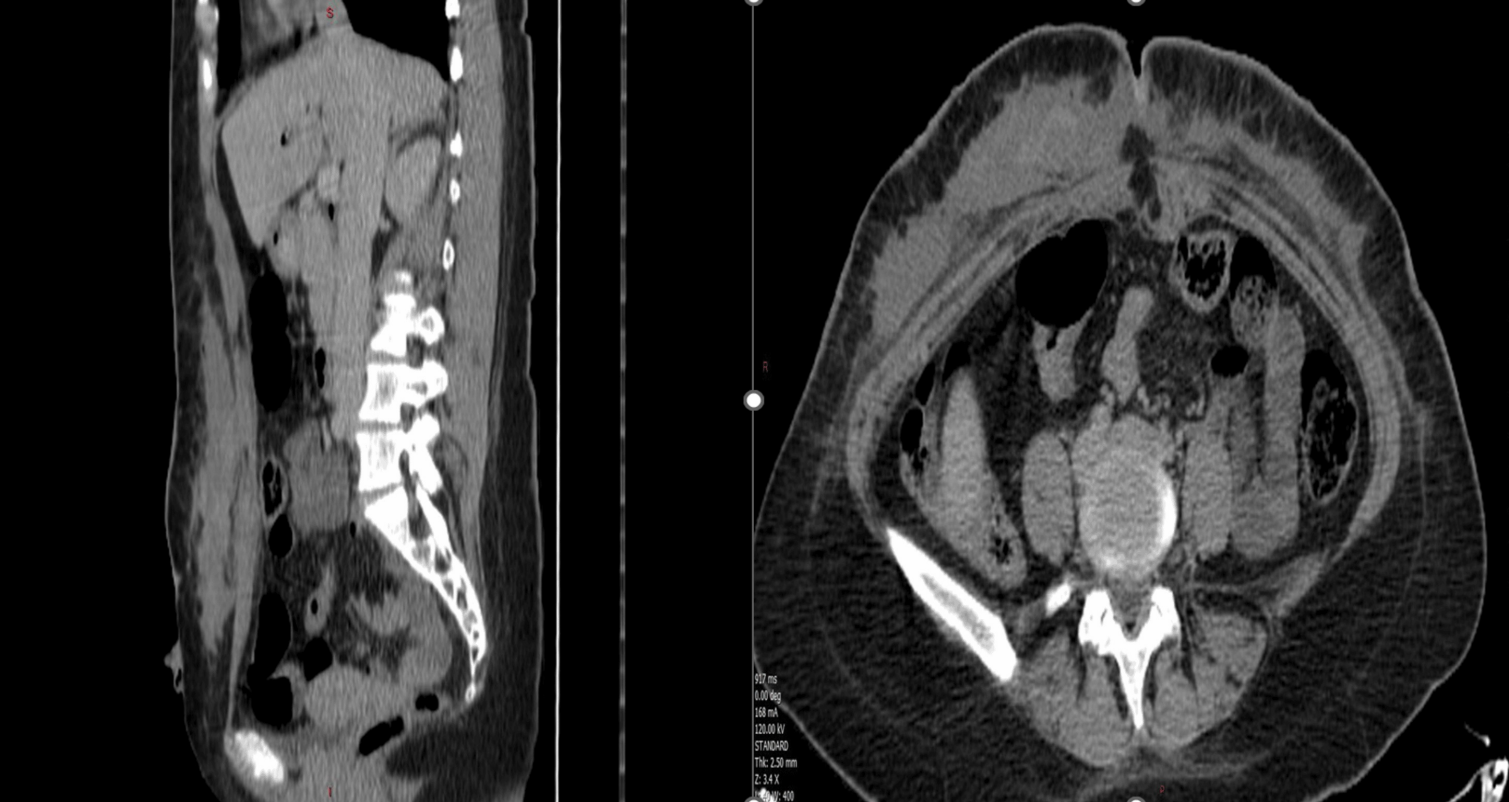
Abdominopelvic CT scan showing extended hematoma and signs of necrotizing fasciitis.

Also, she started to have bloody oozing and secretions around the umbilicus. Blood analysis showed a hemoglobin level of 6.2 g/dL, a white blood cell (WBC) count of 8,830 cells/dL, neutrophils at 88%, and a C-reactive protein (CRP) level of 202 mg/L. Other laboratory values were within normal limits. Based on these results, the patient had a Laboratory Risk Indicator for Necrotizing Fasciitis (LRINEC) score of 6, placing her in the intermediate risk category for necrotizing fasciitis. This, combined with her clinical presentation, raised a strong suspicion of necrotizing fasciitis. An urgent thoraco-abdominopelvic computed tomography (CT) scan was ordered and showed a 23 x 25 x 2.5 cm hematoma in subcutaneous fat of the anterior abdominal wall extending from sternal xyphoid until pubic area, and an anterior defect of linea alba non-communicating with the peritoneal cavity with no intraperitoneal anomaly, dermal thickening, increased soft-tissue attenuation, and inflammatory fat stranding (Figure 2).

Thus, based on the clinical scenario, labs, and imaging, the diagnosis of hematoma and necrotizing fasciitis was confirmed.

**Figure 3:**
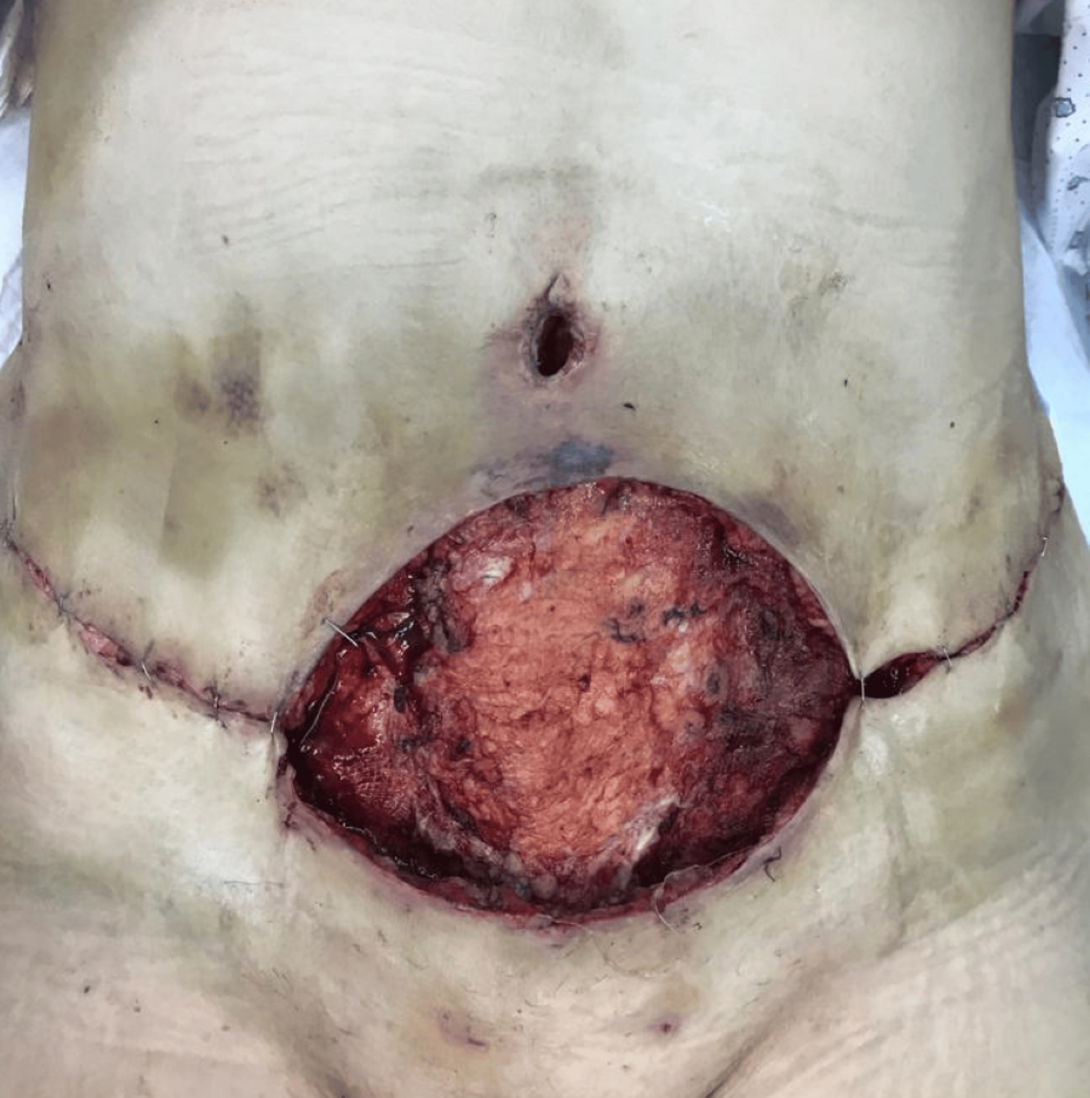
The appearance of the wound after the first session of debridement.

Right away, an urgent surgical exploration was done (within one hour of her presentation), and an important necrosis of subcutaneous tissue and deep fat and thrombosis of subjacent vessels were seen, confirming the diagnosis of necrotizing fasciitis. Evacuation of the hematoma, minimal excision of the ischemic skin and subcutaneous tissue of the upper edge of the wound, debridement of the fascia and the fat of the abdominal wall, with release of diastasis recti plication, were performed with a skin defect on the medial part of the upper flap. Moreover, the wound was cleaned and left open with a wet-dry dressing placed on it (Figure 3).

Post-operatively, the patient was clinically well and stable.

Monomicrobial *E. coli* germs were positive from the abdominal wound culture. Hence, we are reporting this rare case of *E. coli* necrotizing fasciitis post abdominoplasty combined with liposuction with some possible hypotheses and causes.

According to the antibiogram, IV ceftriaxone and IV clindamycin were started (instead of the broad spectrum) as urine and blood cultures were negative.

**Figure 4:**
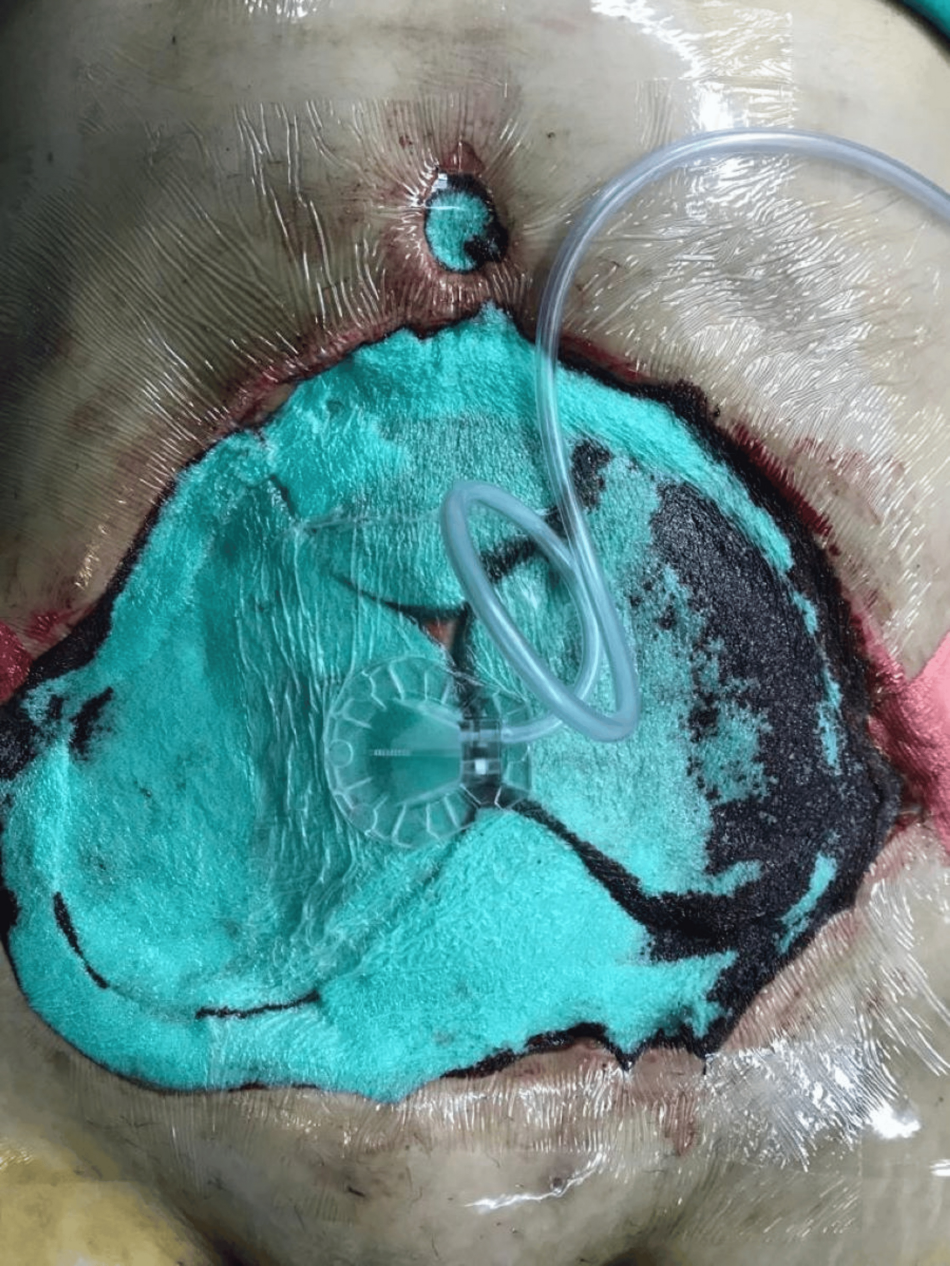
Implementation of negative pressure wound therapy.

**Figure 5:**
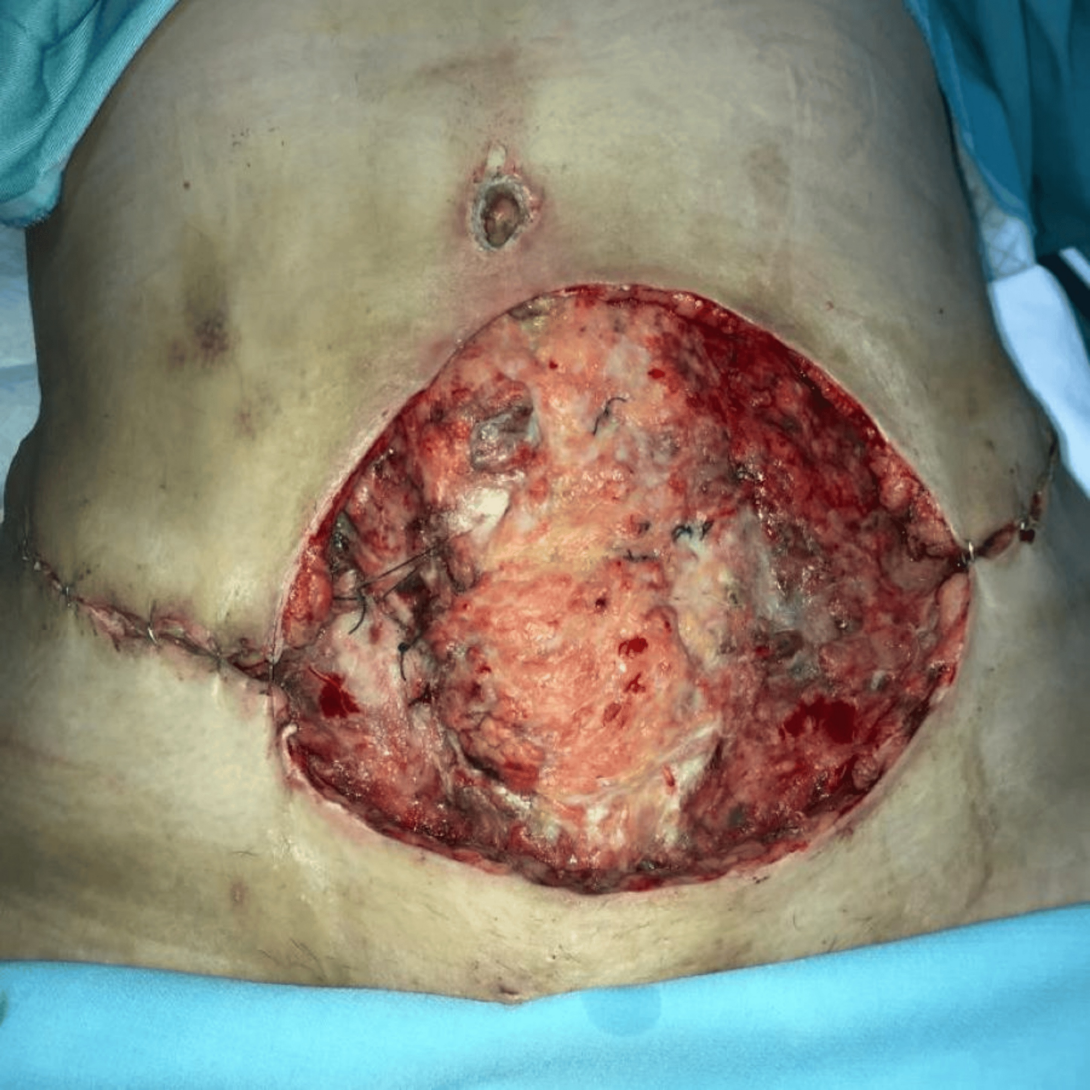
The appearance of the wound after the second session of debridement.

**Figure 6:**
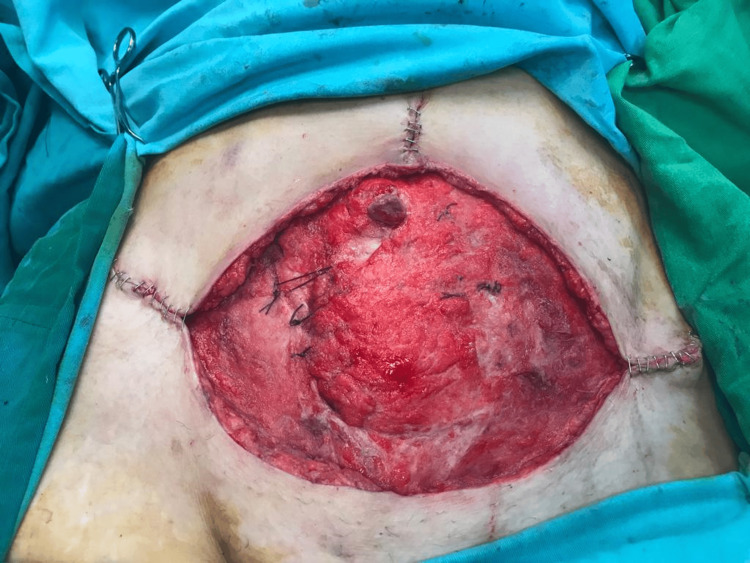
The appearance of the wound after four sessions of debridement and negative pressure wound therapy.

Furthermore, a negative pressure wound therapy (NPWT) was applied (-137 mm Hg) (Figure 4), with four sessions of surgical debridement over a duration of 12 days (Figures 3, 5, 6).

Good granulation tissues were observed with a negative wound culture. The defect was reduced to 6 x 4 cm, followed by a secondary closure using a full-thickness skin graft. Finally, the patient was discharged home in good condition.

## Discussion

Abdominoplasty or tummy tuck remains a top choice among aesthetic surgery procedures. This popularity originates from its different applications and outcomes. Aging individuals desire a youthful abdominal profile, mothers who have recently given birth aim to restore their pre-pregnancy bodies, and bariatric patients look to remove excess skin following huge weight loss. Ultimately, the goal is to remove unwanted skin and fat while tightening abdominal muscles for a more defined body [1]. This surgery can be combined with liposuction in many cases. Abdominoplasty and/or contouring surgery was classified by Matarasso into four types: type 1, liposuction alone; type 2, limited abdominoplasty; type 3, modified abdominoplasty; and type 4, standard abdominoplasty with liposuction [10].

In their literature review, Gilardi et al. found that the most performed cosmetic surgery procedures were abdominoplasty, breast augmentation, liposuction, gluteal augmentation, and foreign body injections (overall, body contouring was the most common category of cosmetic surgeries) [11].

Many complications can occur after abdominoplasty/liposuction surgeries, such as seroma [12], infections which are found to be the second most common complication following abdominoplasty, with an estimated incidence between 1% and 3.8%, hematoma (1.3%), and systemic complications such as thromboembolism (0.8%) [7,12,13]. Other complications, such as flap necrosis due to insufficient irrigation and seromas, also increase the risk of infection [14]. Additionally, in their review, Hafezi and Nouhi showed that wide liposuction has been associated with the danger of ischemia or flap necrosis [15].

Many studies found that male sex, diabetes, increasing age, high body mass index at the time of the surgery, a longer time interval between their initial bariatric surgery and abdominoplasty, and longer operations were associated with a higher complication rate [7,16,17]. In our case, the patient was a young, non-diabetic female and a light smoker, but had a BMI of 28, which may have contributed as a risk factor for infection. Moreover, the overall complication rate for patients undergoing post-bariatric surgery, such as body contouring, ranges from 20% to 66% in the literature [18].

As we mentioned before, one of the most devastating complications is infection. Data from the International Society of Aesthetic Plastic Surgery (ISAPS) showed that abdominoplasty ranked as the fifth most common aesthetic surgery overall following breast augmentations, liposuctions, blepharoplasties, and rhinoplasties [19]. And despite that, abdominoplasty carried a significantly higher risk of infection compared to all others, followed by breast augmentations, liposuctions, and gluteal augmentations [11].

Furthermore, combined procedures, in particular abdominoplasty with liposuction, significantly increase the risk of complications such as infections (3.8%), as compared to abdominoplasty alone (3.1%) [7]. In addition, performing liposuction alongside abdominoplasty increases the risk for ischemia, flap necrosis, and wound dehiscence [15].

Complications related to cosmetic procedures can be categorized according to the Clavien-Dindo classification, which stratifies adverse events based on the type of intervention required, ranging from minor deviations from the normal postoperative course not requiring pharmacological or invasive treatment (grade I), to complications requiring medications such as antibiotics or blood transfusions (grade II), interventions under local or general anesthesia (grade IIIa and IIIb, respectively), life-threatening events requiring intensive care due to single or multi-organ failure (grade IVa and IVb), and ultimately, patient death (grade V) [20].

Gilardi et al. found 92 cases of patients with a grade II infective complication. According to other reports, 188 patients had a grade III infective complication. The review identified nine life-threatening infections (grade IV) with no cases of grade I or grade V infections. Moreover, 223 cases of surgical site infections (SSIs) were reported, along with 42 cases of abscesses, 17 cases of wound dehiscence, 10 cases of cellulitis, six cases of sepsis, and four cases of NF [11]. The exact cause of the high infection rate remains unclear, although several contributing factors can be speculated upon. These factors include the large open dissection spaces that can create a favorable environment for bacterial colonization and subsequent infection [21]. Also, early postoperative discharge with drains in situ, as reported in several cases, could provide a potential entrance for wound infection [22].

Multiple procedures realized simultaneously, malnutrition, obesity, tobacco use, diabetes, and poor hand hygiene represent important risk factors for developing postoperative abdominal infection [7-9]. A case series by Manassa et al. demonstrated that smoking nearly triples the risk of infection, with an infection rate of 12.7% in smokers compared to 5% amongst non-smokers [23].

In cases where infectious complications such as necrotizing fasciitis occur, they may be further classified into four types: type I, the most common, is polymicrobial and often involves organisms such as *E. coli*, *Pseudomonas*, and *Bacteroides*; type II is typically monomicrobial, involving group A streptococcus or sometimes *Staphylococcus aureus*; type III is caused by gram-negative marine-related bacteria like *Vibrio* and *Aeromonas* species; and type IV is fungal, seen in immunocompromised patients with *Candida* or *Zygomycetes* infections [24].

The most common isolated germs in infections encountered after abdominoplasty are mainly the inhabitants of the normal skin bacterial flora, regrouping *Staphylococcus aureus*, *Staphylococcus epidermidis*, and *Streptococcus pyogenes* [8]. Besides, the most predominant etiologic agents in patients who underwent surgery in the Dominican Republic were non-tuberculous mycobacteria (NTM) and *Mycobacterium abscessus*, *Mycobacterium chelonae*, and *Mycobacterium* *fortuitum* [25].

Cases of fungal infections complicating cosmetic surgery are very rare. Rodríguez et al. reported a sole case of *Saksenaea erythrospora*-related mucormycosis developed after combined breast augmentation and abdominoplasty [26]. This occurrence is uncommon in immunocompetent and healthy patients [11,26].

Exceptionally rare cases of *Escherichia coli* and *Enterococcus faecalis* infections post abdominoplasty or liposuction were described [22,27-29].

Several hypotheses can be proposed to explain the *E. coli* infection that can complicate some cosmetic surgeries. The first one is probably explained by poor hand hygiene in some patients.

Additionally, *E. coli *and various germs have the potential to be transmitted through either direct or indirect contact. Teams involved in perioperative care may contact items such as door handles, surgical patient carts, computer keyboards, or other equipment in the operating suite, thereby transferring contaminants to the patient and surgical field. Consequently, maintaining proper hand hygiene is crucial when working with patients [30]. Moreover, good hand hygiene minimizes the chances of transmitting endogenous organisms from the patient and exogenous organisms from other patients, the healthcare team, and the operating room environment [31].

Besides the potential infection risk presented by non-scrubbed personnel, there is also a probability of microbial transmission through sterile gloves, leading to a possible infection. Also, surgical gowns can be contaminated during surgeries [32].

Current evidence endorses the practice of surgical hand scrubs for a duration ranging from three to five minutes at least, coupled with the use of double gloves [33-35].

Evangelista et al. found that coagulase-negative *Staphylococcus* (gram-positive cocci), *Pseudomonas* species (spp), and *E. coli* are the primary microorganisms identified on surgical instruments following clinical usage. *E. coli* was present in 44% of the analyzed materials, and it was also the most isolated gram-negative rod [36].

To note that surgical tools need to undergo a set of consecutive and interconnected steps, such as cleansing, drying, evaluation of integrity and functionality, preparing, and sterilizing, to enable their safe reuse [36].

Microbiological analysis done by Evangelista et al. found that the load of *E. coli* and its adhesion over the surgical instruments gradually rise over time, reaching a level of 105 CFU/cm^2^ following a 24-hour period of contamination. Also, biofilm initiation occurs after two hours. Therefore, the effectiveness of routine cleaning protocols is demonstrably decreased by delayed pre-processing. This delay exacerbates the challenges associated with the cleaning procedure, both in terms of removing a huge microbial load and the cohesive force exerted by the biofilm of *E. coli* arranged on the material's surface. Thus, the importance of pre-cleaning coupled with immediate transport for subsequent processing is noted [36]. Additionally, research suggests that post-sterilization contamination can also increase the incidence of serious deep SSIs [37].

Another hypothesis is the *E. coli* translocation. Many studies showed the ability of microorganisms and toxins typically found in the gastrointestinal (GI) tract to translocate to locations outside the intestines in a few cases. Furthermore, it was recorded that among the translocated bacteria, *E. coli* was the most frequently found [38,39]. Many studies showed that bacterial translocation (BT) can happen shortly after abdominal surgery, as early as two hours post operation. This translocation can be due to many factors, including visceral angiospasm triggered by anesthesia and trauma, bowel ischemia, a decrease in oxygen due to blood loss, the generation and release of vasoactive factors, prostaglandins, and inflammatory cytokines, and ischemia-reperfusion injury. These effects can be observed even when there is no direct surgical injury to the bowels since the inflammatory cytokines, anoxemia, and blood loss can happen in any major surgery, including abdominoplasty and/or body contouring [39-43].

Here are some recommendations for reducing the risk and rate of complications: (1) non-smoking healthy (non-diabetic) patients with normal weight (BMI), aged less than 55 years, and doing a single procedure in a hospital or accredited surgical center rather than office-based surgical suites [7,16]. (2) Shorter time interval between the initial bariatric surgery and abdominoplasty [16]. (3) Undermining the abdominal flap by extreme caution, preserving the perforator vessels above the umbilicus, and a proper amount of tissue resection are the safeguards for having a viable flap and prevention of segmental necrosis [15,16]. (4) Prophylactic antibiotics before the incision [44]. (5) Deep venous thrombosis prophylaxis [45,46]. (6) Perioperative, intraoperative, and postoperative nurses and medical teams should use best practices for surgical skin antisepsis to prevent inadvertent transfer of contaminants to patients throughout the surgical procedure and to prevent any possible ischemia-reperfusion injury [32,43]. (7) Proper hand hygiene. (8) Early detection and treatment of complications.

Diagnosis

Clinical findings and symptoms are important for the early diagnosis of NF. They can be divided into two groups, early and advanced symptoms [47]. Moreover, primary or idiopathic NF manifests without an obvious causative factor or point of entry of microorganisms. In contrast, secondary NF occurs due to a recognized underlying cause and arises through skin lacerations, contusions, burns, bites, subcutaneous injections, or surgical incisions. Erythema, pain, local warmth, skin induration, and edema are the most frequent early signs. In the acute and fulminant form of the NF, the patient is in a critical condition, displaying symptoms of severe septic shock and multiple organ dysfunction syndrome (MODS), along with widespread necrosis in the soft tissues. On the other hand, the subacute NF progresses slowly over days or weeks in an indolent clinical course [47,48].

Early surgical exploration of the infection site, combined with microbiological and histopathological analysis, is the gold standard for making and confirming an early NF diagnosis [49]. Grayish necrotic deep fascia, a lack of resistance of normally adherent muscular fascia to blunt finger dissection ("Finger test”), lack of bleeding from the fascia, and the presence of dishwater pus are the most crucial indicators and signs for the NF diagnosis during surgical procedure [50,51].

Hence, NF remains a clinical diagnosis. While imaging has a limited role, a CT scan can be useful for assessing the extent of the disease, assisting in surgical planning, and ruling out alternative conditions. Further, the quick results of CT scans, especially for an emergent NF evaluation, may offer advantages over magnetic resonance imaging (MRI). It is crucial to note that in severely toxic cases, treatment should not be postponed for imaging procedures [52].

Treatment

Today, the widely accepted protocol for treatment is as follows: (1) resuscitate the patient in shock; (2) begin with broad spectrum antibiotics, which cover polymicrobial infection; (3) early debridement of all necrotic tissue and culture from the wound; (4) additional debridement’s should be done every 24 to 48 hours until the infection is controlled; (5) appropriate antibiotic therapy adjusted to the causative germ(s); (6) reconstructive surgeries if needed [53].

NPWT has been reported to draw out exudates, cover wounds securely, promote angiogenesis, limit bacterial growth, and improve the survival and take of skin grafts and flaps [50,51,54,55]. Once the wound has stabilized, fresh granulation tissue has formed, and is free of any signs of acute infection, we move to the reconstruction stage. This stage includes various techniques, ranging from simple to complex, to restore the form and function of the affected area [51].

## Conclusions

NF is a rare but highly lethal complication of cosmetic surgeries. Key factors to reduce the risk include performing surgeries on healthy, non-smoking patients under 55 years of age with a normal weight, minimizing the time between bariatric and aesthetic surgeries, maintaining good hand hygiene, and exercising extreme caution while undermining the abdominal flap. Rare cases of *E. coli* infections post abdominoplasty or liposuction have been reported, due to poor hand hygiene, contamination by perioperative care teams, delayed cleaning of surgical instruments, post-sterilization contamination, and *E. coli* translocation.
